# Sulfur Compounds in Regulation of Stomatal Movement

**DOI:** 10.3389/fpls.2022.846518

**Published:** 2022-03-11

**Authors:** Zirong Ren, Ru-Yuan Wang, Xin-Yuan Huang, Yin Wang

**Affiliations:** ^1^Institute of Ecology, College of Urban and Environmental Sciences and Key Laboratory for Earth Surface Processes of Ministry of Education, Peking University, Beijing, China; ^2^State Key Laboratory of Crop Genetics and Germplasm Enhancement, College of Resources and Environmental Sciences, Nanjing Agricultural University, Nanjing, China

**Keywords:** abscisic acid, guard cell, hydrogen sulfide, stomatal movement, sulfur compounds, sulfur dioxide

## Abstract

Sulfur, widely present in the soil and atmosphere, is one of the essential elements for plants. Sulfate is a dominant form of sulfur in soils taken up by plant roots. In addition to the assimilation into sulfur compounds essential for plant growth and development, it has been reported recently that sulfate as well as other sulfur containing compounds can also induce stomatal movement. Here, we first summarized the uptake and transport of sulfate and atmospheric sulfur, including H_2_O and SO_2_, and then, focused on the effects of inorganic and organic sulfur on stomatal movement. We concluded all the transporters for different sulfur compounds, and compared the expression level of those transporters in guard cells and mesophyll cells. The relationship between abscisic acid and sulfur compounds in regulation of stomatal movement were also discussed.

## Introduction

Sulfur is an essential macronutrient required for plant growth and development. Sulfur is a constituent of the amino acids cysteine (Cys) and methionine (Met) which are necessary for the synthesis of proteins and serve as precursors of important cofactors and sulfur containing secondary metabolites (Thomas et al., 2000; Mugford et al., 2011). The metabolites of sulfate assimilation and metabolism have important effects on plant growth, development, environmental response, resistance to biological and abiotic stress, crop quality and yield (Zhao et al., 1999; Hell et al., 2010; Gironde et al., 2014). Recent studies have suggested that sulfur containing compounds may also play a role in regulation of stomatal movement. Stomata are micro pores mainly found on leaf surface of terrestrial plants, controlled by two guard cells. CO_2_ and H_2_O exchanging through stomatal pores makes stomatal movement a key process for photosynthesis and drought resistance. In the present review, we focused on the transporters and summarized different sulfur compounds on regulating stomatal movement in details.

## Sulfate Uptake in Roots and Long-Distance Transport

**FIGURE 1:**
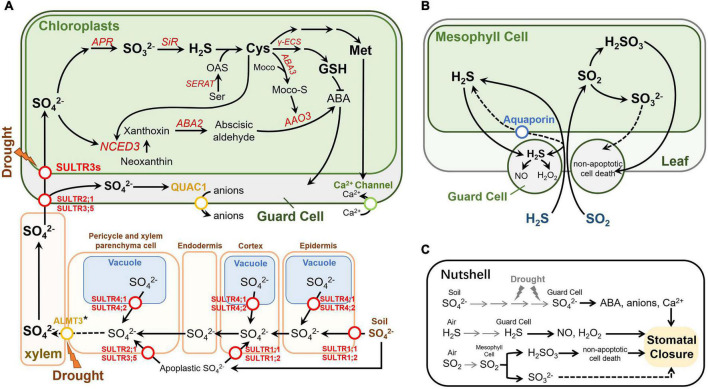
Effects of sulfur on stomatal movement in *Arabidopsis thaliana*. **(A)** Sulfate transport and metabolism in Arabidopsis under drought conditions. The sulfate transport function of ALMT3 marked with an asterisk was found in poplar trees (Malcheska et al., 2017). This figure partially refers to Takahashi et al. (2011) and Batool et al. (2018). **(B)** Transport and metabolism of exogenous H2S and SO2 in Arabidopsis. **(C)** A nutshell of the effect of sulfur on stomatal movement. Circles on the arrowed lines indicate transporters, and different transporter families are shown in different colors. Enzymes are indicated in red italics. Dashed arrows indicate disputable or putative pathways. Gray arrows indicate the transportation of the sulfur. Abbreviations not mentioned: ALMT3, putative aluminum-activated malate transporter 3.

There are two sulfur uptake pathways in plants, including the sulfate uptake in roots and atmospheric sulfur entry pathway through stomata. Plants take up sulfur from soils by roots mainly in form of sulfate through sulfate transporters (SULTRs). In plants, genes encoding sulfate transporters are divided into four distinct subfamilies (*SULTR1* to *SULTR4*) according to the similarity of the protein sequences (Vatansever et al., 2016). In the genome of *Arabidopsis thaliana*, there are eleven SULTR members and the functions of most of *SULTRs* have been comprehensively studied. In *Arabidopsis*, AtSULTR1;1 and AtSULTR1;2 are two high-affinity sulfate transporters responsible for sulfate uptake in roots (Figure 1A). AtSULTR1;2 is involved in the sulfate uptake in roots under sulfate sufficient condition, while AtSULTR1;1 is responsible for the absorption of sulfate under sulfate limitation condition (Hideki et al., 2000; Naoko et al., 2002; Rouached et al., 2008). *AtSULTR1;2* is expressed in the root epidermal and cortical plasma membranes, and is co-localized with *AtSULTR1;1* (Hideki et al., 2000; Naoko et al., 2002). Although sulfate deficiency induced the expressions of both *AtSULTR1;1* and *AtSULTR1;2*, the induction of *AtSULTR1;1* is much stronger than that of *AtSULTR1;2* (Barberon et al., 2008; Rouached et al., 2008).

After entering root cells, sulfate could be transported into the vacuole for storage or translocated to shoots for assimilation in plastids. The flux and allocation of sulfate in plants depends on the demand of plants organs and cells for sulfate, as well as sulfur status of the whole plant (Rennenberg and Herschbach, 2014; Spicer, 2014). The root-to-shoot or shoot-to-root long-distance transport of sulfur in high plants takes place in the vascular system which consists of xylem and phloem (Rennenberg et al., 1979). The existence of sulfate in phloem sap and phloem exudate has long been demonstrated (Toshiyuki et al., 1990). In rice, sulfate deprivation for 7 days results in a decrease in sulfate concentration in the phloem sap, which suggests that sulfate can be transferred between shoots and roots (Kuzuhara et al., 2000). In *Arabidopsis*, the loading sulfate into sieve tube of phloem is mediated by AtSULTR1;3 which is mainly expressed in the sieve element-companion cell complexes in the cotyledons and roots (Kuzuhara et al., 2000). In addition to phloem transport, xylem transport is also an important role of sulfate translocation (Yoshimoto et al., 2003). AtSULTR3;5 and AtSULTR2;1 are involved in loading sulfate to xylem parenchyma cell and facilitate the root-to-shoot translocation (Figure 1A; Kataoka et al., 2004a).

Remobilization of sulfate from vacuole is critical for maintenance of sulfur homeostasis in plants, particularly under sulfate limited condition. In *Arabidopsis*, it has been demonstrated that tonoplast-localized AtSULTR4;1 and AtSULTR4;2 facilitate the sulfate efflux from the vacuoles (Figure 1A) evidenced by the increase of vacuolar sulfate concentration in the *atsultr4;1 atsultr4;2* double knockout mutant (Kataoka et al., 2004b). Interestingly, although both *AtSULTR4;1* and *AtSULTR4;2* are induced by sulfur deficiency, they display differential sulfur-dependent expression pattern in roots. *AtSULTR4;2* can increase accumulation in respond to sulfate limitation while *AtSULTR4;1* constitutively expresses under different sulfate conditions. Expression of *AtSULTR4;1* in the *sultr4;1 sultr4;2* double knockout mutant can rescue its phenotype indicating that *AtSULTR4;1* plays a primary role in maintaining intracellular sulfate redistribution and homeostasis (Kataoka et al., 2004b; Martinoia et al., 2007). However, whether AtSULTR4;1 and AtSULTR 4;2 participate in the sulfate efflux from vacuole in guard cells is not clear.

Once translocation from roots to shoots, sulfate is transported into plastids for assimilation. In *Arabidopsis*, AtSULTR3 family proteins are involved in transport of sulfate into chloroplasts (Figure 1A). Simultaneous knockout of all five members of *AtSULTR3* reduced sulfate accumulation in chloroplasts by more than 50% compared to the wild type (Chen et al., 2019). In plastids, sulfate is first converted to adenosine-5′-phosphosulfate (APS) by ATP sulfurylase (ATPS) (Logan et al., 1996; Thomas et al., 2000), and further reduced to sulfide in a two-step reduction reaction catalyzed by APS reductase (APR) and sulfite reductase (SiR) (Olivier et al., 2007; Khan et al., 2010a). In the last step of sulfate primary metabolism, cysteine (Cys) is produced by condensation of sulfide and *O*-acetylserine (OAS) catalyzed by OAS (thiol)lyase (OASTL) (Heeg et al., 2008). Cys serves as a precursor for the biosynthesis of Met, GSH and other sulfur containing compounds (Ravilious and Jez, 2012). In parallel, APS also can be phosphorylated to PAPS, which is involved into sulfation reaction in secondary metabolism as a precursor of active sulfate (Mugford et al., 2009, 2011).

## Atmospheric Sulfur Uptake and Metabolism

In addition to absorb sulfate *via* roots, plants are also able to utilize foliary-absorbed sulfur gases as directly sulfur source, such as hydrogen sulfide (H_2_S) and sulfur dioxide (SO_2_) (Figure 1B; Herschbach et al., 1994; Sue et al., 2002; Aghajanzadeh et al., 2016). Atmospheric sulfur gases are derived from natural source and anthropogenic source. H_2_S and SO_2_ emitting by volcanic and geothermic activity are the main natural sulfur source while SO_2_ is the main anthropogenic sulfur source releasing from industrial processes and human life (Garrec, 1984; Stern, 2005). The kinetics of SO_2_ entry into plant leaves is correlated with stomatal conductance and SO_2_ level in the atmosphere (Noland and Kozlowski, 1979; By et al., 1996). SO_2_ is soluble in water phase in mesophyll cells, and reacts with water to release hydrogen ion (H^+^) and generate hydrogen sulfite (HSO_3_^–^). HSO_3_^–^ can be directly reduced and assimilated into organic sulfur compounds in chloroplasts, or further oxidized into sulfate before entering the sulfur assimilation pathway (Noland and Kozlowski, 1979; De Bruyn et al., 1995). Different from SO_2_, the conductivity of mesophyll cells to H_2_S is largely determined by its metabolic rate in plants and the H_2_S level in atmosphere. Due to the poor solubility of H_2_S in water, H_2_S can be dissociated into H^+^ and hydrogen sulfide ion (HS^–^) in the atmosphere (By et al., 1996; Stuiver and De Kok, 2001; Lee et al., 2011).

The inorganic SO_2_ or H_2_S entry into plant leaves could be further assimilated into organic sulfur through sulfate assimilation pathway. Plants exposed to SO_2_ or H_2_S gases significantly increase the thiol content and change thiol composition in shoots (Aghajanzadeh et al., 2016; Ausma et al., 2021). In *Arabidopsis*, a short-term fumigation with 0.25 μl l^–1^ H_2_S strongly increase the concentrations of cysteine and glutathione by 20 and 4 times, respectively (Riemenschneider et al., 2005). However, the content and composition of glucosinolate in *Brassica juncea* and *Brassica rapa* were not affected by SO_2_ or H_2_S exposure regardless of sulfur sufficiency or deprivation (Aghajanzadeh et al., 2015). While SO_2_ and H_2_S exposure as sulfur compensation, can actually make foliar absorb more sulfur gas than sulfate-sufficient condition, but the absorption of sulfur nutrition in shoot does not affect the accumulation of transcript caused by sulfate limitation in roots, while their exposure can alleviated the up-regulated of *APR*, rather *SULTR1;1*, *SULTR1;2* or *OASTL* (Stuiver and De Kok, 2001; Aleksandra et al., 2008; Birke et al., 2015). Although SO_2_ and H_2_S exposure may affect sulfate uptake in roots, the expression levels of *SULTRs* are independent on their exposure (Aleksandra et al., 2008). To be sure, SO_2_ and H_2_S is also a well-known toxic gas that can cause harm to plants at deleterious concentrations which may vary from plant species and environmental conditions (Thompson and Kats, 1978; Malhotra and Khan, 1984). It is controversial for determining what degree of foliar absorption contributes its toxification or helpfulness on account of variability of the growing environment and nutrient needs of different plants (Amaral et al., 2006; Yang et al., 2006; Lisjak et al., 2013).

## Effects of Inorganic Sulfur on Stomatal Movement

### Sulfate

Sulfate is the main inorganic sulfur form in plants, which has been found to induce stomatal closure under drought stress (Goodger et al., 2005; Ernst et al., 2010; Batool et al., 2018). Recent studies suggested that sulfate itself is not able to induce stomatal closure as knockout of key enzymes in sulfate assimilation pathway, such as SiR and Ser acetyltransferase (SERAT), abolishing the sulfate induced stomatal closure (Batool et al., 2018). Upon drought stress, plants increase the translocation of sulfate from root to shoot through xylem (Goodger et al., 2005). The accumulation of sulfate in shoots induces abscisic acid (ABA) synthesis through two paralleled pathways in *Arabidopsis*. In the first pathway, inorganic sulfate is reduced to organic sulfur compound Cys through sulfate assimilation pathway (Figure 1A). After that, using Cys and molybdenum cofactor (MoCo) as substrates, MoCo-S is synthesized by molybdenum cofactor sulfatase ABA3, thereby activating ABCISIC ALDEHYDE OXIDASE3 (AAO3) (Xiong et al., 2001; Wollers et al., 2008). Activated AAO3 could catalyze the final step in ABA biosynthesis (Seo et al., 2004). In the second parallel pathway, increased sulfate and synthesized Cys enhance the transcription of 9-*cis*-epoxycarotenoid dioxygenase 3 (*NCED3*), which is a drought-stress-induced isoform and provides a substrate precursor for AAO3, thus contributing to ABA biosynthesis (Nambara and Marion-Poll, 2005; Endo et al., 2008; Malcheska et al., 2017; Batool et al., 2018). The ABA induced by these two processes regulates the stomata closure through a series of signal transduction process (Blatt, 2000; Kim et al., 2010; Abhilasha and Choudhury, 2021). Moreover, the application of extracellular sulfate could directly regulate the R-Type anion channel QUICK ANION CHANNEL 1 (QUAC1), which was also known as aluminum-activated malate transporter 12 (ALMT12). The *quac1* mutant fails to close stomata under the application of sulfate, indicating that QUAC1 is required for sulfate induced stomatal closure (Meyer et al., 2010; Malcheska et al., 2017).

**FIGURE 2:**
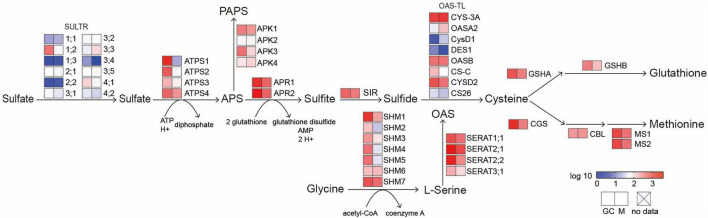
A diagram of sulfur metabolic pathways. Heat map indicates the expression levels of genes involved in sulfate transport and assimilation in Guard cells and mesophyll cells. *SULTR*, sulfate transporter; *ATPS*, ATP sulfurylase; *APR*, adenosine-5′-phosphosulfate reductase; *APK*, adenosine-5′-phosphosulfate (APS) kinase; *SIR*, sulfite reductase; *OAS-TL, O*-acetylserine (thiol) lyase; *SERAT*, serine acetyltransferase; *SHM*, serine hydroxymethyltransferase; *GSHA*, gamma-glutamylcysteine synthetase; *GSHB*, glutathione synthetase B; *CGS*, cystathionine gamma-synthase; *CBL*, cystathionine beta-lyase; *MS*, methionine synthase. GC, guard cells; M, mesophyll cells. Expression value derived from Arabidopsis eFP browser was normalized by log10 (http://bar.utoronto.ca/efp/cgi-bin/efpWeb.cgi).

The ion transport process after sulfate sensing in plants has not been studied in detail. Recently, it is speculated that sulfate may trigger a signal to close stomata in guard cells and vasculature. In guard cells, the completion of the above-mentioned processes requires to transport sulfate into plastids. This is because the reduction of sulfate and the beginning of ABA biosynthesis both occur in the plastid (Khan et al., 2010b). Members of the AtSULTR3 subfamily, preferentially expressed in the chloroplast membrane of leaves, are considered to be one of the most important sulfate transporters (Cao et al., 2013; Chen et al., 2019). Remarkably, transcription of four out of the five AtSULTR3 members (3;1, 3;2, 3;4, and 3;5) is enriched in guard cells (Bauer et al., 2013). The expression of *AtSULTR 2;1*, a AtSULTR3;5-activated sulfate transporter located on the plasma membrane, was also significantly higher than that of mesophyll cells (Leonhardt et al., 2004). Taken together, these results indicate that sulfate could be transported to guard cells even more efficiently than to mesophyll cells. The expression level of *AtSULTR3;3* in mesophyll cells and guard cells is not significantly different, which may be due to the functionally redundant of SULTR3s, resulting in the total contribution of individual members higher than 100% (Cao et al., 2013). It does not seem necessary to increase the expression level of all SULTR3 members in guard cells. The unchanged expression levels of ATP-binding cassette (ABC) and triose-P/P-translocator (TPT), which are also plastid-localized backup sulfate transport systems, may also because the redundancy of SULTR3s (Hampp and Ziegler, 1977; Chen et al., 2003). Moreover, Chen et al. (2019) mutated all five members of the SULTR3 subfamily and found that in the quintuple mutants, in addition to the significant reduction in chloroplast sulfate absorption, the downstream Cys and ABA were also significantly reduced after the application of exogenous sulfate, and stomatal closure was also abolished. This defect could be compensated by adding sulfide or Cys to induce the stomatal closure (Cao et al., 2014). These results indicate that SULTR3s are also an important part of sulfate-induced stomatal signal transduction. Similarly, APR2, an enzyme that catalyzes the key step of sulfate reduction and tightly regulates the sulfate assimilation pathway, is also enriched in guard cells (Loudet et al., 2007; Bauer et al., 2013). The *apr2* mutant accumulates less ABA than wild-type plants when external sulfate is applied (Cao et al., 2014), demonstrating that guard cells may be able to efficiently complete the sulfate assimilation process and produce ABA. This is supported by the fact that most of genes involved in sulfate assimilation are expressed in guard cells (Figure 2). Sulfate can also induce the transcription of *NCED3* in guard cells, thereby accumulating ABA and promoting stomatal closure (Figure 1C; Malcheska et al., 2017).

In the vasculature, sulfate or Cys can also induce *NCED3* transcription, suggesting that sulfate or Cys may also induce the synthesis of ABA in the vasculature. Consistent with this, the *NCED3* transcription level and NCED3 protein level also increased significantly in the vasculature of drought-stressed plants (Endo et al., 2008). However, the detailed process remains to be determined.

### Hydrogen Sulfide

The effect of H_2_S on stomatal movement has been well discussed in a recent review (Liu and Xue, 2021). Despite some controversy, most studies have shown that exogenous application of H_2_S can induce stomatal closure mainly in molecular form (Lisjak et al., 2010; Jin et al., 2013; Chen et al., 2020). Several phytohormones and signaling molecules, such as ABA, NO, hydrogen peroxide (H_2_O_2_), and 8-mercapto-cGMP, are also involved in H_2_S-induced stomatal movement (Figure 1C; Jin et al., 2013; Chen et al., 2020; Zhang et al., 2021). H_2_S also acts as a signal, interacts with various phytohormones (including ABA, ethylene, salicylic acid, and jasmonic acid) and other signaling molecules, such as NO and ROS, and regulates stomatal movement in response to biotic and abiotic stress (Garcia-Mata and Lamattina, 2001, 2010; Hou et al., 2013; Deng et al., 2020; Zhang et al., 2021). Furthermore, H_2_S mediates post-translation modification of protein through phosphorylation and S-persulfidation to control ABA-dependent stomatal closure. The persulfidation of DES1, a pivotal enzyme producing H_2_S, was induced by ABA accompanied by synthesis of ROS (Shen et al., 2020; Zhang et al., 2021). Persulfidation-base modification change the structure of the key kinase protein SNF1-RELATED PROTEIN KINASE 2.6 (SnKR2.6) in ABA signaling pathway, resulting in enhanced kinase activity, and the phosphorylation modification level at key sites of SnKR2.6 protein can positively regulate H_2_S-mediated sulfhydrylation modification (Chen et al., 2021).

Notably, H_2_S, as a highly lipophilic gaseous signaling molecule, can freely pass through the phospholipid membrane for signal transmission (Li and Moore, 2008). It is speculated that some channel proteins may contribute to H_2_S transport and increase the efficiency of H_2_S permeation (Figure 1B). Because the structure of H_2_S is as similar as of H_2_O, there is a hypothesis that aquaporins can promote the absorption of H_2_S (Lee et al., 2005). Aquaporins are membrane channels widely found in plants, animals, and microbe, which promote the passage of water and small neutral molecules through cell membranes (Maurel et al., 1993). By analyzing the crystal structure of the aquaporin-M (ApqM) in *Methanothermobacter marburgensis*, Lee et al. (2005) found that one of its pore geometries could easily accommodate H_2_S, therefore speculating that AqpM could promote the absorption of H_2_S. However, through the study of the aquaporin in a sulfide-reducing bacteria *Archaeoglobus fulgidus* (AfAQP), an evolutionarily close protein of AqpM, it was found that AfAQP cannot promote the absorption of H_2_S (Mathai et al., 2009). The homologous proteins of AfAQP also exist in plants (such as Aquaporin TIP 3;2 and NIP1;2 in *Arabidopsis*), but the H_2_S transport of these aquaporins has not been studied.

### Sulfur Dioxide

Sulfur dioxide could be absorbed through stomata and dissolved in the cytoplasm, and is hydrolyzed to sulfurous acid (H_2_SO_3_), bisulfite ion (HSO_3_^–^) and sulfite ion (SO_3_^2–^) according to the pH value (Figure 1B). However, the effect of SO_2_ on stomatal movement in plants is controversial. Different studies reported that SO_2_ could decrease (Winner and Mooney, 1980; Olszyk and Tibbitts, 1981; Rao et al., 1983), no change (Van der Kooij et al., 1997; Hu et al., 2014), or increase the stomatal aperture (Mansfield and Majernik, 1970; Black and Black, 1979; Biscoe et al., 2006). The different results may be due to different sulfur dioxide concentrations or different plant species. Recently, it was proposed that SO_2_ mainly induces stomatal closure in the form of H_2_SO_3_ (Figure 1C). The stomatal movement induced by SO_2_ is different from that induced by O_3_ and CO_2_, and is mainly caused by non-apoptotic cell death that does not depend on pH changes (Figure 1C; Ooi et al., 2019). This result is inconsistent with the previous study that SO_2_-induced stomatal movement depends on ABA accumulation (Taylor et al., 1981). Moreover, low concentration of SO_2_ could also induce stomatal opening under light and meanwhile stimulate cell death in guard cells in *Arabidopsis* (Ooi et al., 2019).

### Sulfite

The sulfite in plants is mainly derived from the reduction of sulfate, the degradation of Cys and methionine, and the hydrolysis of atmospheric SO_2_ in the apoplastic mesophyll (Yi and Meng, 2003; Hansch and Mendel, 2005). Sulfite could be further reduced to sulfide by SiR or oxidized to sulfate by peroxisome-localized sulfite oxidase (SO). Long-term effects of sulfite on stomatal movement is usually associated with SO_2_. Therefore, similar to SO_2_, the effects of sulfite on stomatal movement, transpiration, and water loss in sulfite-applied plants is not conclusive (see SO_2_ section). Recently, overexpression of APR2 or knock down of SO by RNAi which both increase sulfite accumulation could induce stomatal opening and increase water loss, suggesting sulfite more like promote the stomatal opening rather closure (Bekturova et al., 2021).

## Effects of Organic Sulfur Compounds on Stomatal Movement

Several organic sulfur compounds could also induce stomatal closure. Batool et al. (2018) found that the application of Cys could reduce stomatal aperture in *Arabidopsis*, and the sulfate-induced stomatal closure is dependent on the biosynthesis of Cys. GSH is involved in ABA-induced stomata closure (Okuma et al., 2011). Decreasing GSH level in guard cells in the GSH biosynthesis deficient mutant *cad2-1* or inhibition of GSH biosynthesis enhanced ABA-induced stomatal closure (Figure 1A). Overexpression of gamma-glutamylcysteine synthetase (γ-ECS), a rate-limiting enzyme in GSH biosynthesis, significantly reduced the stomatal aperture and density (Lu et al., 2021). Moreover, exogenous application of L-methionine (L-Met) has been shown to enhance stomatal closure by activating Ca^2+^ channels and generation of reactive oxygen species (ROS) (Kong et al., 2016).

## Conclusion and Future Perspectives

Whether from soil or atmosphere, sulfur compounds mainly cause stomatal closure, like stress signal, in an ABA dependent or independent pathway. Except for sulfate, the transporters of H_2_S and SO_2_ are still unrevealed. The underlying mechanisms of the relationship between drought stress and sulfate accumulation in guard cells need to be investigated in the future.

## Author Contributions

ZR and R-YW wrote the manuscript. X-YH and YW reviewed and edited the manuscript. All authors have discussed and approved the submitted version of the manuscript.

## Conflict of Interest

The authors declare that the research was conducted in the absence of any commercial or financial relationships that could be construed as a potential conflict of interest. The reviewer YX declared a shared affiliation with several of the authors, R-YW and X-YH, to the handling editor at the time of the review.

## Publisher’s Note

All claims expressed in this article are solely those of the authors and do not necessarily represent those of their affiliated organizations, or those of the publisher, the editors and the reviewers. Any product that may be evaluated in this article, or claim that may be made by its manufacturer, is not guaranteed or endorsed by the publisher.
